# Supplementary material for: Stochastic activity in low-rank recurrent neural networks
Source: PLoS Comput Biol. 2025 Aug 18;21(8):e1013371. doi: 10.1371/journal.pcbi.1013371 (PMC12413093; doi:10.1371/journal.pcbi.1013371)
Supplement: Attachment — Submitted filename: rebuttal.pdf [file pcbi.1013371.s001.pdf]

Reviews are in blue; responses in black; reported manuscript text in red.

Additional Editor Comments: We suggest adding a brief discussion in the Discussion section on the relationship between learning and emergent connectivity. In particular, it would be illuminating to consider how the dimensionality of inputs during learning—low-dimensional versus high-dimensional—may influence the rank of the resulting connectivity matrix.

We thank the Editor for the suggestion. We have added to the Discussion:

Another domain where our results may open new avenues is the study of learning in biological and artificial recurrent neural networks. Low-rank connectivity matrices are particularly relevant in this context, as synaptic updates often take a low-rank form [31, 23], and in some cases, the final learned connectivity also retains a low-rank structure [26, 27]. Most learning algorithms adjust synaptic connectivity based on estimates of network activity and its covariance structure [31, 23, 53, 22]. In this work, we showed that both activity and covariance depend sensitively on the dimensionality and geometry of stochastic inputs. As a result, in plastic recurrent networks, different noise structures in the inputs are expected to produce not only distinct activity patterns but also fundamentally different connectivity profiles. Consistent with these predictions, recent work has demonstrated that, in recurrent networks trained via gradient descent, the presence of stochastic inputs can qualitatively alter the solution found by the learning algorithm [25]. For instance, noise can bias learning toward configurations that better align with experimental observations [54], or induce additional low-rank connectivity components that suppress variability along task-relevant directions [42]. However, this study did not systematically investigate how the amplitude and geometry of input noise shape the learned connectivity. Our framework offers several predictions about this relationship, which remain to be tested and explored in future work. For example, since the rank of the learned connectivity is linked to the dimensionality of network activity, we expect it to scale with the dimensionality of external stochastic inputs. Moreover, we have seen that any learned low-rank connectivity term effectively suppresses variability along specific directions, which could be exploited by the learning algorithm for robust decoding of information. We finally remark that, although trained networks often rely on non-linear dynamics, our linear framework remains broadly applicable, as linearization around fixed points is a common and powerful strategy for analyzing such systems [55, 26].

We also note the following minor points for your attention:

In the paragraph following Equation (2), the manuscript states: "Our analysis focuses on the activity generated by this model in the stationary regime, which is reached provided that the real part of all eigenvalues of W is less than 1." It may help readers if you clarify that this condition ensures the real part of the eigenvalues governing the dynamics is negative, thereby stabilizing the system.

The use of in Equation (11) may be confusing, given that is already used for network activity earlier in the manuscript. Consider using a different symbol to avoid ambiguity.

We thank the Editor for his careful reading. We have implemented those changes.

Reviewer 1: In this work the authors study the dynamics of low-rank linear recurrent neural networks driven by stochastic inputs. The dimensionality of the input is varied and the interplay between input dimensionality and weight structure is studied. This leads to some important effects on the dimensionality of the dynamics themselves, which are shown to be high-dimensional even in cases where the weights are low-rank. This contrasts with previous studies by the authors and others, and they do a good job explaining why the differences occur.

I found the modeling interesting and the paper very well-written. Overall, I think the paper is well-suited for PLOS CB.

One main weakness, in my opinion, is the restriction to just linear dynamics. Can similar qualitative behavior can occur in nonlinear networks? Another weakness is that the connections to biology aren't at the forefront of the paper. For the most part it concerns the mathematical results, although a nice application to very simple E/I networks appears at the end of the results. The emphasis is on computation over biology, so if the biological connections could be made stronger in the discussion or elsewhere, that would help.

We thank the reviewer for his positive assessment.

We agree with the reviewer on the importance of considering non-linear dynamics. Importantly, although our analysis is based on linear dynamics, it can be also used to describe the behavior of non-linear networks near stationary states. This is because, in such regimes, the dynamics can be effectively linearized, and the linearization process modifies the connectivity matrix in a low-rank manner. As a result, our findings remain relevant for understanding network responses in many non-linear settings, at least as a first-order approximation. We now clarify this point in the Discussion:

We finally remark that, although trained networks often rely on non-linear dynamics, our linear framework remains broadly applicable, as linearization around fixed points is a common and powerful strategy for analyzing such systems [55, 26].

Moreover, preliminary numerical investigations suggest that several of the phenomena described in this work may qualitatively generalize to non-linear recurrent networks. (One relevant simulation can be found in Supp. Fig. 7–8 of the parallel study by Wan and Rosenbaum, arXiv 2025.) That said, one of the main advantages of low-rank recurrent neural networks is that they allow for analytical tractability even in fully non-linear regimes. We are therefore optimistic that systematic and quantitative extensions of our results to the case of non-linear networks can be developed, and we see this as a promising direction for future work.

We also agree with the reviewer's comment regarding biological relevance. As a historical note, this work was directly inspired by experimental observations – specifically, studies examining variability in the activity of neurons across multiple brain areas [62]. We discuss this line of research and its connection to our analysis in the final paragraph of the Discussion. In parallel work, which we expect to make publicly available soon, we apply some of the theoretical tools developed in this manuscript to investigate that specific biological question in great depth, including explicit connections to and re-analyses of experimental data. Since the two projects address distinct objectives and are largely self-contained, we have chosen to present them in separate manuscripts.

In response to the reviewer's advice, we have incorporated a couple of additional sentences into the manuscript, discussing activity dimensionality in both models and experiments.

This results in covariance spectra that are largely flat (Fig. 4A-D), but exhibit distinct values at the low and high ends. This profile contrasts with the gradually decaying spectra typically observed in networks with high-rank connectivity [18]. Therefore, networks with high- and low-rank connectivity driven by high-dimensional inputs can be distinguished based on the profile of their covariance spectra – an activity property that can be easily estimated from experimental data [40].

and

Substantial differences in activity dimensionality have also been reported in experimental studies, with low-dimensional activity often observed in frontal and associative cortices [5, 6], but not necessarily in sensory areas [40]. Our findings indicate that, within the context of models with low-rank connectivity, these differences can be reconciled by positing that associative regions are mostly characterized by positive connectivity overlap (leading to attractor-like dynamics), while sensory regions exhibit more negative overlap (leading to autoencoder-like activity).

Besides the inclusion of a link to the code that ran the experiments, I have otherwise minor suggestions for the authors to improve the paper.

We have added a link to the github repository.

The parallel study by Wan and Rosenbaum that looked at low-rank linear dynamics is mentioned in the discussion. The work here should be compared and contrasted with that in more detail. Please clarify how "their mathematical framework differs" and the "results are qualitatively consistent". A full paragraph in the discussion is likely needed, and Wan and Rosenbaum might be referenced in the introduction.

We have expanded the section of the Discussion where we discuss this study. We now write:

Recent parallel work by Wan and Rosenbaum [10] analyzed input responses in large low-rank networks with strong recurrent connectivity. In that framework, the entries of the low-rank connectivity scale with network size as $1/N$, in contrast to our setting where the scaling is $1/N$. This difference has an important implication: the connectivity in [10] only yields stable dynamics in the case of negative eigenvalues, whose magnitude typically diverges with the network size. Within that regime, the authors report high-dimensional activity and suppression of inputs along the directions defined by the connectivity vectors. Our results are qualitatively consistent with these findings. In our framework, networks with negative overlap ρ_{mn} exhibit negative eigenvalues; as shown in Fig. 3C–D, the corresponding dynamics are primarily suppressive, giving rise to high-dimensional activity. Compared to [10], our framework accommodates a broader range of scenarios. In particular, our analysis reveals that even for positive eigenvalues, some form of suppression can still be observed: while activity is amplified along certain directions, it is concurrently suppressed along others, resulting in intermediate dimensionality.

I'm not sure I see Section 4.1 as being very helpful as part of the main text. Most of the mathematics in here seems more suited to the appendix. There's a lot of notation introduced to describe the filtered signals that doesn't appear again. On the other hand, the last two paragraphs of that section are helpful for interpreting Eqns (7-9); it makes sense to keep those in the main text.

Following the reviewer's suggestion, we have removed one equation, and significantly streamlined the derivation contained within the first part of the section.

Minor points below

Abstract: "remains however not fully understood", awkward

page 3, 4th para: sentence "While the dependence..." has em-dashes formatted wrong

page 6, 1st para: This dimensionality measure goes back much further than ref 3. It was originally introduced in papers by Yule (studying linguistics) and later Simpson (studying ecology) in the 1940s and is equivalent to the Renyi 2-entropy.

page 6, 2nd para: "estimated" should be "computed" since there is no approximation here.

page 6, 3rd para: 2nd sentence was confusing to me and would be better phrased like: "Within the m-u plane, the eigenvectors $v_\pm$ define the principle components of the activity fluctuations."

pg 7, sec 4.1, 2nd para: Define second covariance term

page 8, 1st para: The notation $\hat{\chi}_i(\Lambda)$ hides the t-dependence. This isn't a transform into a timescale variable but still a time-dependent function. I would keep the t-dependence and and also be explicity that the averages $\langle ... \rangle$ are over the time variable. Earlier, these are used to be averages over input realizations.

page 11, eqn 27: superscript notation λ^r looks like a power; in fact this is intermixed in this equation

with variables raised to powers. To clarify where you are using superscripts, I suggest writing $\lambda^{(r)}$, etc.

We thank the reviewer for careful reading. We have changed the text to incorporate all the suggestions.

(In page 8, 1st paragraph, angular brackets still denote averages over input realizations, evaluated in the stationary state, for which $t \to \infty$. We now clarify it – apologise for the confusion.)

Reviewer 2: In this paper Mastrogiuseppe et al. extend the low-rank dynamical framework developed initially by the first author in a very logical and valuable way. I found this paper adds a crucial piece to this body of work, while being thorough and interesting. Overall I don't have much to add, only a few very minor comments and suggestions, mostly on exposition or details.

We thank the reviewer for the positive feedback.

Section 6: I would move the definition of sum and difference vectors from the fig legend to the text, for clarity. In the figure, perhaps consider putting an indication of E = 0; I = 90 (e.g. on the plot axes), or a little schematic to the left (e.g. a vector diagram or so, but this might be overkill), just to help visualization. In the second-to-last paragraph, maybe include n and m as parentheticals to remind the reader of the relationships where you say "For input vectors strongly aligned with the difference direction (n) ... Instead, for inputs strongly aligned with the sum direction (m)...". In paragraph 3, add a citation(s) in the last sentence beginning "Consistent with previous work..."

We have implemented those changes, thank you.

I didn't recall seeing a link to the published code in the paper, so please include one in the methods (apologies if I just missed it).

We have included a link to the github repository.

A few typos I noticed:

Intro, paragraph 5: "...low-rank RNNs in response to this type of INPUT is ..."

After eq. 5, definition of propagator operator, should (W - 1) be written (W - I)

Pg. 9, first paragraph: "Along OF all other directions, including v_+..." - remove "of"

Fig. 5 caption: "... from simulated activity and THE THE theoretically-estimated ..." (double "the")

Section 6, last sentence: "... similarly to THE rank one case ..."

Section 6, paragraph 3: "... a natural framework for studying INPUT amplification"

We thank the reviewer for pointing out the typos, we have corrected them.

Reviewer 3: The manuscript investigates the role of stochastic, high-dimensional inputs in shaping the dynamics of low-rank networks. This is an important problem to study, since previous papers mainly focused on deterministic low-dimensional inputs to low-rank networks that are unrealistic for modeling brain dynamics. The authors find that high-dimensional inputs could give rise to high-dimensional dynamics depending on the network structure. The manuscript is clearly written, and especially the analytical derivations are thoroughly described and easy to follow. The findings provide new insights

into how the input structure shapes the dynamics of low-rank networks and will be valuable to a broad range of neuroscientists interested in how input and network structure jointly shape neural dynamics. I just have a few questions and minor suggestions outlined below.

We thank the reviewer for the positive assessment.

Questions: 1. How much do the findings related to high-dimensional inputs depend on the fact that all inputs have the same mean and variance? For example, would dependency on m and n change if specific input dimensions are stronger or more variable?

Since we consider linear dynamics, changes in the input mean shift the overall activity levels but do not affect the structure or amplitude of activity fluctuations. As a result, our findings remain unchanged. In particular, our analysis focuses on covariances, which are computed after mean subtraction; these quantities are invariant to changes in the input mean.

In contrast, changes in the variance of the stochastic inputs do affect the results: increasing the variance of one input relative to others effectively reduces the dimensionality of the input. This is because such manipulation corresponds to increasing the magnitude of one column of U relative to the rest. The limit case of one-dimensional input discussed in the manuscript – obtained by setting all columns of U to zero except one – can be viewed as an extreme instance of this type of perturbation. The general case where the columns of U have different magnitude can be understood as an interpolation between the one- and high-dimensional cases we analyze in detail in the manuscript.

2. How do the model predictions change as the rank of the connectivity increases? Would, at a certain rank, the role of input in shaping high-dimensional dynamics vanish, and dynamics become dominated by recurrent components rather than input?

Whether network activity is dominated by external inputs or recurrent connectivity depends on the relative amplitudes of these two components. Importantly, the rank of the connectivity matrix is not inherently linked to its amplitude. In this work, for simplicity, we do not normalize the norm of the recurrent connectivity as the rank increases. As a result, recurrent contributions can grow and potentially dominate over input-driven activity. However, in biological and artificial neural networks, one can plausibly assume the existence of homeostatic mechanisms that constrain the overall strength of synaptic connections, even as their dimensionality increases. Under such constraints, the amplitudes of recurrent and input contributions could remain comparable, allowing both to shape activity to a similar extent.

3. In a real brain, how could one distinguish the role of high-dimensional inputs versus high-rank connectivity in shaping high-dimensional dynamics? Does the proposed theory provide any testable predictions? If such predictions exist, it would be interesting to mention them in the Discussion.

We thank the reviewer for the insightful question. The two scenarios (high-dimensional inputs versus high-rank connectivity) are in fact characterized by very different covariance spectra. The latter can be easily measured in experiments. We now highlight this difference in the manuscript:

This results in covariance spectra that are largely flat (Fig. 4A-D), but exhibit distinct values at the low and high ends. This profile contrasts with the gradually decaying spectra typically observed in networks with high-rank connectivity [18]. Therefore, networks with high- and low-rank connectivity driven by high-dimensional inputs can be distinguished based on the profile of their covariance spectra – an activity property that can be easily estimated from experimental data [40].

Minor suggestions: 1. To make the paper accessible to a broader audience, it would be helpful if authors could provide a summary of the findings in the form of a table or graphical abstract. While the analytical derivations are described in detail, a non-expert audience may prefer to get a general understanding of dependencies without going through the details of the calculations.

2. In figures 2, 4, 5, and 6, it would be helpful to add the variable describing the colorbars directly to the figure, instead of only mentioning it in the caption.

3. If Section 3 only discusses one-dimensional inputs, it would be more informative to change the title of this section to “one-dimensional stochastic inputs”.

4. In the last paragraph of the Discussion about how trial-to-trial variability relates to network structure, the authors could also refer to the following experimental and modeling studies relating trial-to-trial variability to the neural connectivity structure:
- Rosenbaum, R., Smith, M. A., Kohn, A., Rubin, J. E., Doiron, B. (2017). The spatial structure of correlated neuronal variability. Nature neuroscience, 20(1), 107-114.
- Safavi, S., Dwarakanath, A., Kapoor, V., Werner, J., Hatsopoulos, N. G., Logothetis, N. K., Panagiotaropoulos, T. I. (2018). Nonmonotonic spatial structure of interneuronal correlations in prefrontal microcircuits. Proceedings of the National Academy of Sciences, 115(15), E3539-E3548.
- [already mentioned in the introduction] Huang, C., Ruff, D. A., Pyle, R., Rosenbaum, R., Cohen, M. R., Doiron, B. (2019). Circuit models of low-dimensional shared variability in cortical networks. Neuron, 101(2), 337-348.
- Shi, Y. L., Steinmetz, N. A., Moore, T., Boahen, K., Engel, T. A. (2022). Cortical state dynamics and selective attention define the spatial pattern of correlated variability in neocortex. Nature communications, 13(1), 44.

5. For reproducibility purposes, I recommend that authors provide a README for their shared code that describes what each script does and which figures they reproduce.

We thank the reviewer for suggesting the changes. We have implemented them.
